# Racial and ethnic representation in systemic sclerosis clinical trials: a 25-year analysis of ClinicalTrials.gov (1999-2025)

**DOI:** 10.1016/j.ero.2025.11.002

**Published:** 2025-12-08

**Authors:** Phong Nguyen, Yujin Kim, Andrew Price

**Affiliations:** 1 Department of Internal Medicine, William Beaumont Army Medical Center, Fort Bliss, TX, USA; 2 Texas Tech Health Sciences University Center El Paso, Paul L. Foster School of Medicine, El Paso, TX, USA; 3 Department of Rheumatology, William Beaumont Army Medical Center, Fort Bliss, TX, USA

## Abstract

**Objectives:**

This study aimed to quantify reporting of race and ethnicity in systemic sclerosis (SSc) clinical trials, characterise geographic distribution, and assess participant representation relative to US population benchmarks.

**Methods:**

We conducted a cross-sectional analysis of registered SSc trials with enrolment data. Of 699 trials screened, 95 met initial criteria; after excluding 21 terminated and 4 non-SSc studies, 70 completed trials (n = 4956 participants) were analysed. We summarised the prevalence of race/ethnicity reporting and calculated race/ethnicity distributions among participants with available data. US Census estimates were used for contextual comparison. Trials were also grouped by recruiting continent.

**Results:**

Among 70 trials, 39 (55.7%) reported race and 26 (37.1%) reported ethnicity; 25 reported both, while 30 (42.9%) reported neither. Participant-level data were available for 3161 individuals for race (60.0% of all enrollees) and 2255 for ethnicity (47.1%). Among those with race reported, 78.5% were White (n = 2482), 12.7% Asian (n = 400), 4.9% Black or African American (n = 155), 1.4% multiracial (n = 45), 0.6% American Indian/Alaska Native (n = 18), and 0.3% Native Hawaiian/Other Pacific Islander (n = 8); 1.7% were unknown/unreported (n = 53). Of participants with ethnicity data (n = 2335), 10.9% were Hispanic/Latino, 86.6% not Hispanic/Latino, and 2.5% unknown/unreported. Trial activity was concentrated in North America (39.2%) and Europe (36.3%), with additional activity in Asia (16.6%); South America, Oceania, and Africa contributed smaller shares.

**Conclusions:**

Race/ethnicity reporting in SSc trials is incomplete, and available data reveal disproportionate underenrolment of Black and Hispanic/Latino participants relative to US population benchmarks. Standardised reporting and intentional, equity-focused recruitment are needed to enhance representativeness and interpretability of SSc trial findings.

**WHAT IS ALREADY KNOWN ON THIS TOPIC**

• Systemic sclerosis (SSc) disproportionately affects racial and ethnic minorities; African American patients tend to have earlier onset, more severe phenotypes, and poorer outcomes.
• Prior assessments suggest clinical trials across many diseases often underrepresent minority groups, and demographic reporting is inconsistent.
• For SSc specifically, a comprehensive, registry-based quantification of who is enrolled, and how consistently race/ethnicity are reported, has been limited.

**WHAT THIS STUDY ADDS**

• From 699 registered SSc trials (1999-2025), 70 interventional trials with 4956 participants met inclusion criteria for demographic analysis.
• Demographic reporting was incomplete; race reported in 39 trials (60.0% of participants) and ethnicity in 26 trials (47.1% of participants); 42.9% of trials reported neither race nor ethnicity.
• Compared with 2020 US Census benchmarks, Black/African American and Hispanic/Latino participants were underrepresented, while Asian and White participants were overrepresented.
• The study highlights structural features likely contributing to imbalances, including site geography, and optional demographic reporting.

**HOW THIS STUDY MIGHT AFFECT RESEARCH, PRACTICE OR POLICY**

• This study supports standardised, mandatory reporting of race and ethnicity in SSc trials to enable monitoring, subgroup analyses, and transparency.
• It motivates inclusive site networks beyond North America/Europe, pragmatic eligibility, and participant-friendly designs to broaden access.
• It informs funders and regulators to incorporate measurable diversity targets and accountability into trial planning and approvals.
• It guides clinicians and guideline developers on the generalizability limits of existing evidence and the need to interpret treatment effects cautiously for underrepresented groups.
• It establishes a baseline for longitudinal tracking of SSc trial equity, enabling evaluation of future policy and infrastructure changes.

Alt-text: Unlabelled box

## INTRODUCTION

Systemic sclerosis (SSc) is a rare autoimmune connective tissue disorder marked by widespread fibrosis and vascular abnormalities [1]. Significant variation in disease prevalence, severity, and clinical phenotype has been documented across racial and ethnic groups. For example, African American patients are more likely to present with early-onset, diffuse cutaneous disease and experience poorer survival outcomes than White patients [2,3]. Although SSc is frequently reported among White women, population-based data indicate high incidence among Black women, suggesting underestimation of disease burden in this group [4]. These differences underscore the importance of diverse representation in clinical trials.

Despite longstanding awareness of health disparities in SSc, the racial and ethnic composition of clinical trial cohorts has not been comprehensively assessed. Adequate representation in clinical research ensures that treatment efficacy and safety are generalisable and equitable. It also facilitates identification of subgroup-specific responses and helps address disparities in disease burden and access to emerging therapies.

This study aimed to evaluate racial and ethnic representation in SSc clinical trials registered on ClinicalTrials.gov between 1999 and 2025. By examining demographic reporting practices and comparing trial populations with US Census benchmarks [5], this analysis identifies critical gaps in equity and offers recommendations to improve inclusivity in rare disease research.

## METHODS

### Data source

ClinicalTrials.gov was queried for studies registered between 1999 and 2025 that investigated SSc as a primary condition. Inclusion criteria were as follows: (1) interventional design, (2) enrolment of human subjects, and (3) available enrolment data. Trials with terminated status due to poor enrolment or lacking relevance to SSc were excluded.

### Data extraction

Extracted variables included National Clinical Trial identifier, enrolment size, completion/termination status dates, race and ethnicity, and geographic distribution. Race was categorised as White, Asian, Black/African American, American Indian/Alaska Native, Native Hawaiian/Other Pacific Islander, multiracial, and unknown. Ethnicity was classified as Hispanic/Latino, Not Hispanic/Latino, or unknown.

### Statistical analysis

Descriptive statistics were used to summarise trial characteristics and demographic variables using counts and percentages. At the participant level, we pooled race/ethnicity shares by weighting each trial by its enrolment and compared these against 2020 US Census benchmarks using participation-to-population ratios and absolute differences. Trials without demographic reporting were included in trial-level summaries but excluded from participant-level estimates. Visualisation tools were used to illustrate disparities in reporting and representation.

## RESULTS

### Study selection

**Figure 1:**
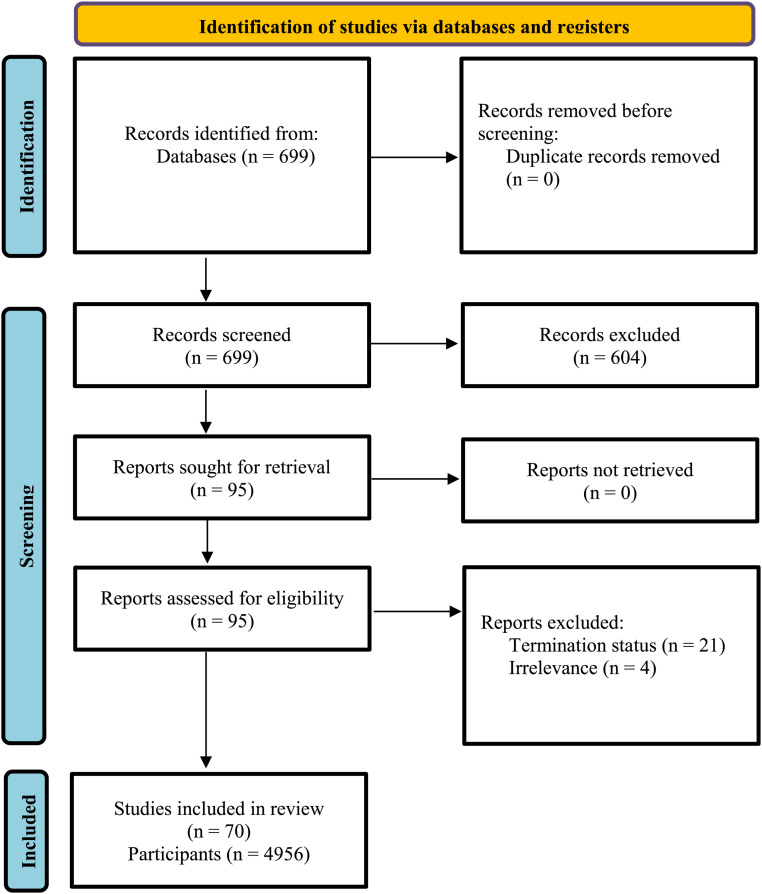
Flow diagram of the study selection process.

Of 699 registered trials with enrolment data, 95 met initial inclusion criteria. Following the exclusion of 25 trials (21 terminated and 4 unrelated to SSc), a final cohort of 70 completed trials with 4956 participants was analysed (Fig 1).

### Study characteristics

Among the 70 SSc trials analysed, 39 studies (55.7%) reported race data. Reporting of ethnicity was more limited, with 26 trials (37.1%) providing this information. While 25 trials reported both race and ethnicity, 30 trials (42.9%) did not report any demographic data. Participant-level reporting covered 3161 individuals for race (60.0% of all enrollees) and 2255 for ethnicity (47.1%) of 4956 participants. These data are provided in the Table.

**Table:** Reporting of race and ethnicity data at the trial and participant levels

Demographic reporting category	Trials, n (%)	Participants, n (%)
Race data		
Reported	39 (55.7)	3161 (60.0)
Not reported	31 (44.3)	1495 (40.0)
Ethnicity data		
Reported	26 (37.1)	2255 (45.5)
Not reported	44 (62.9)	2701 (54.5)
Combined demographic data		
Both race and ethnicity reported	25 (35.7)	—
Race only reported	14 (20.0)	—
Ethnicity only reported	1 (1.4)	—
No demographic data reported	30 (42.9)	—
Totals	70 (100.0)	4956 (100.0)

Combined categories are not available at the participant level.

### Demographic reporting

**Figure 2:**
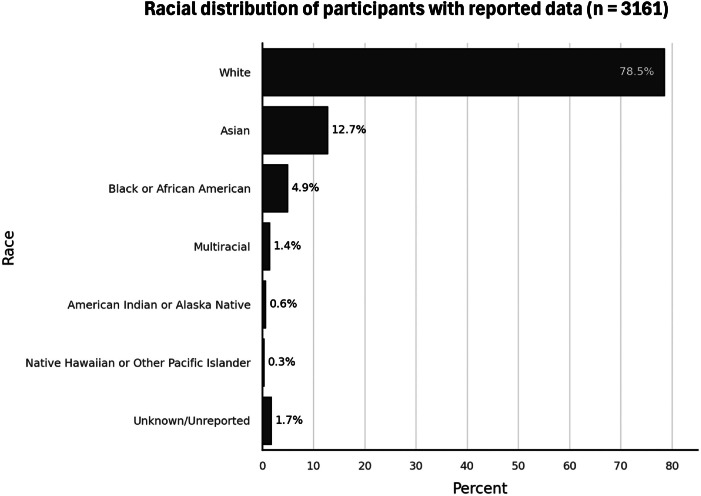
Racial distribution of participants with reported race data (n = 3161).

**Figure 3:**
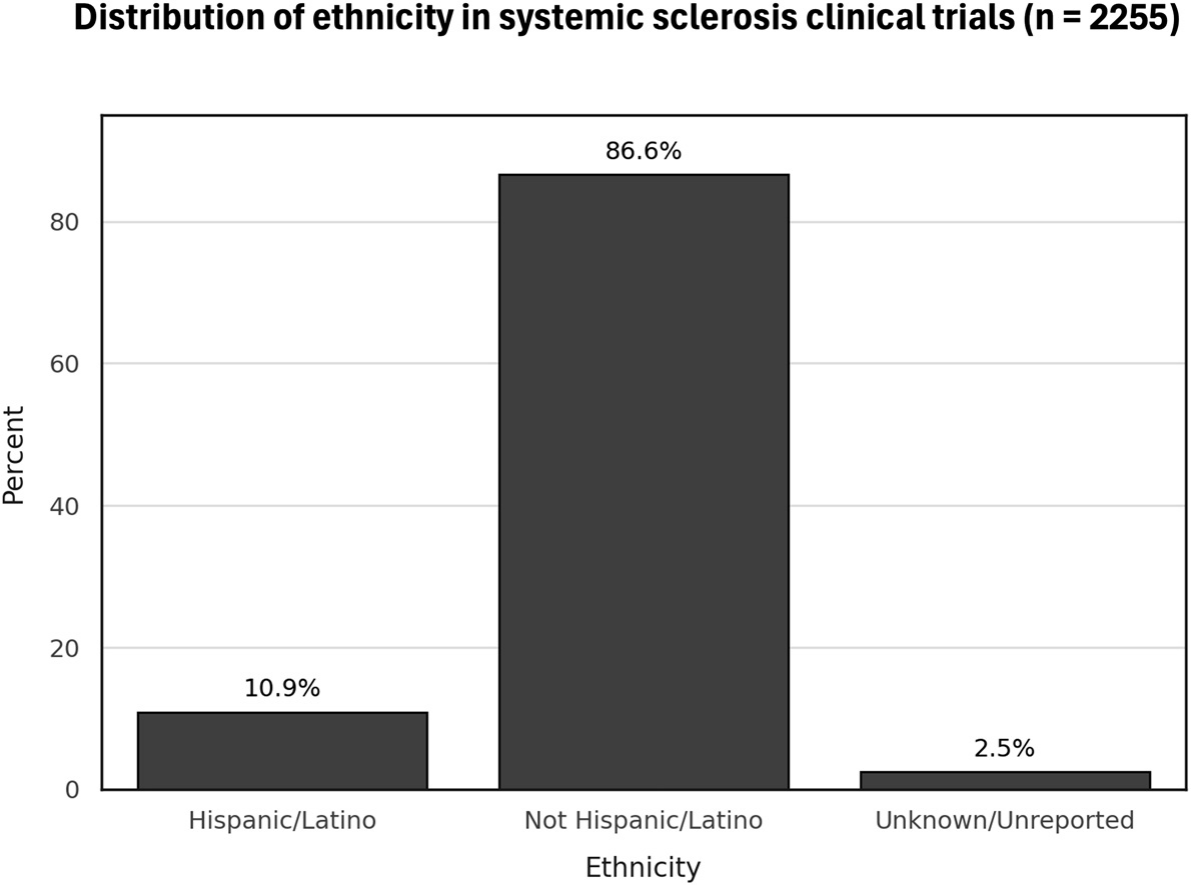
Ethnicity distribution in systemic sclerosis clinical trials (n = 2255).

Of the 3161 participants with reported race data, the majority were White (n = 2482; 78.5%). Asian participants comprised the second-largest group (n = 400; 12.7%), followed by Black or African American individuals (n = 155; 4.9%). Representation from other racial groups was minimal: 45 participants (1.4%) were identified as multiracial, 18 (0.6%) were American Indian or Alaska Native, and 8 (0.3%) were Native Hawaiian or Other Pacific Islander. An additional 53 participants (1.7%) had race data classified as unknown or unreported (Fig 2). Ethnicity data (n = 2335) revealed that 10.9% were Hispanic/Latino, 86.6% were Not Hispanic/Latino, and 2.5% had unknown/unreported ethnicity (Fig 3).

### Geographic distribution

**Figure 4:**
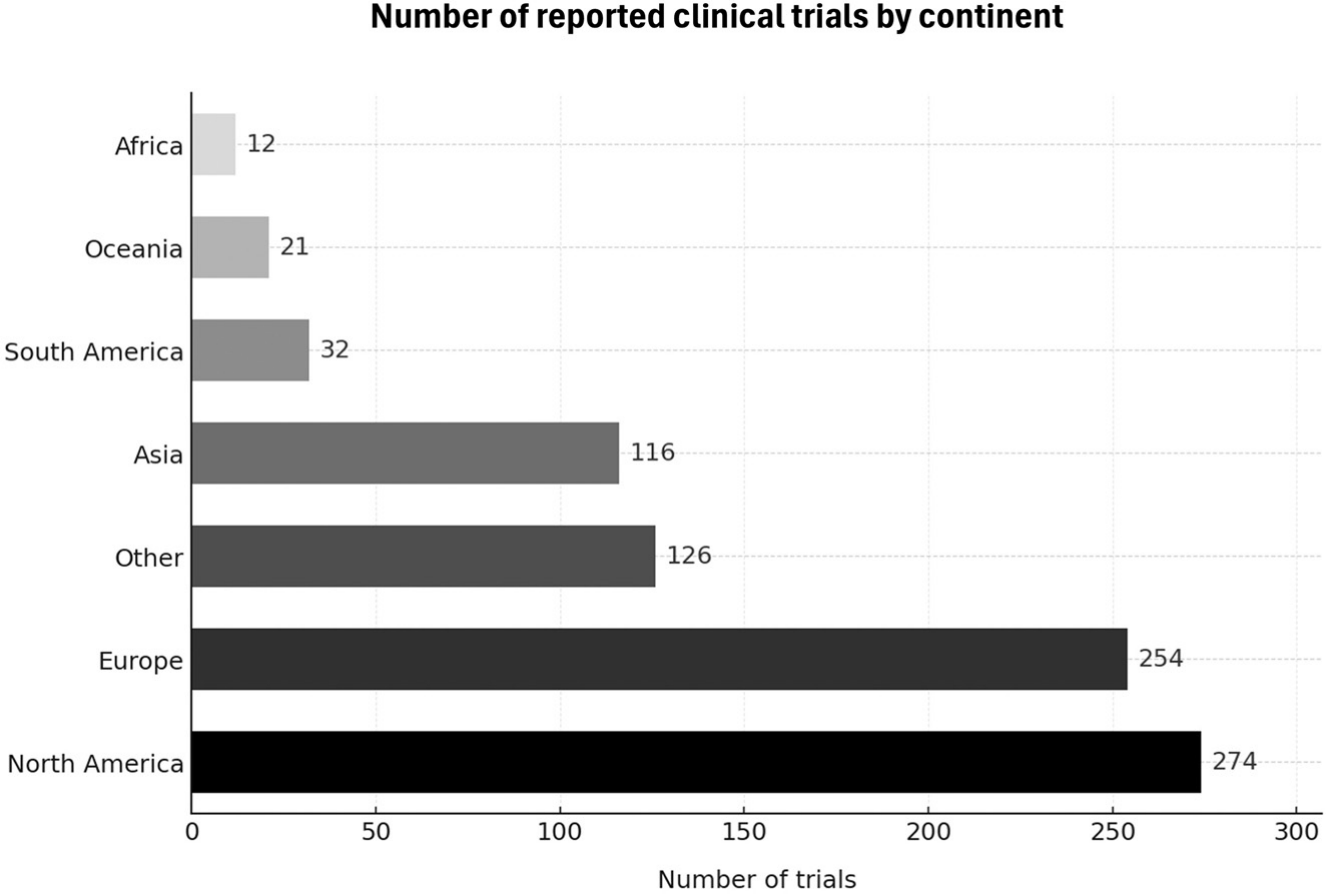
Distribution of clinical trials stratified by continent.

When grouped by continent, North America leads with 274 trials (39.2%), followed by Europe with 254 trials (36.34%). Asia accounts for 116 trials (16.6%), while other categories (unknown or unassigned countries) comprise 126 trials (18.03%). South America, Oceania, and Africa have smaller shares with 32 (4.58%), 21 (3.0%), and 12 (1.72%) trials, respectively (Fig 4).

### Census comparison

**Figure 5:**
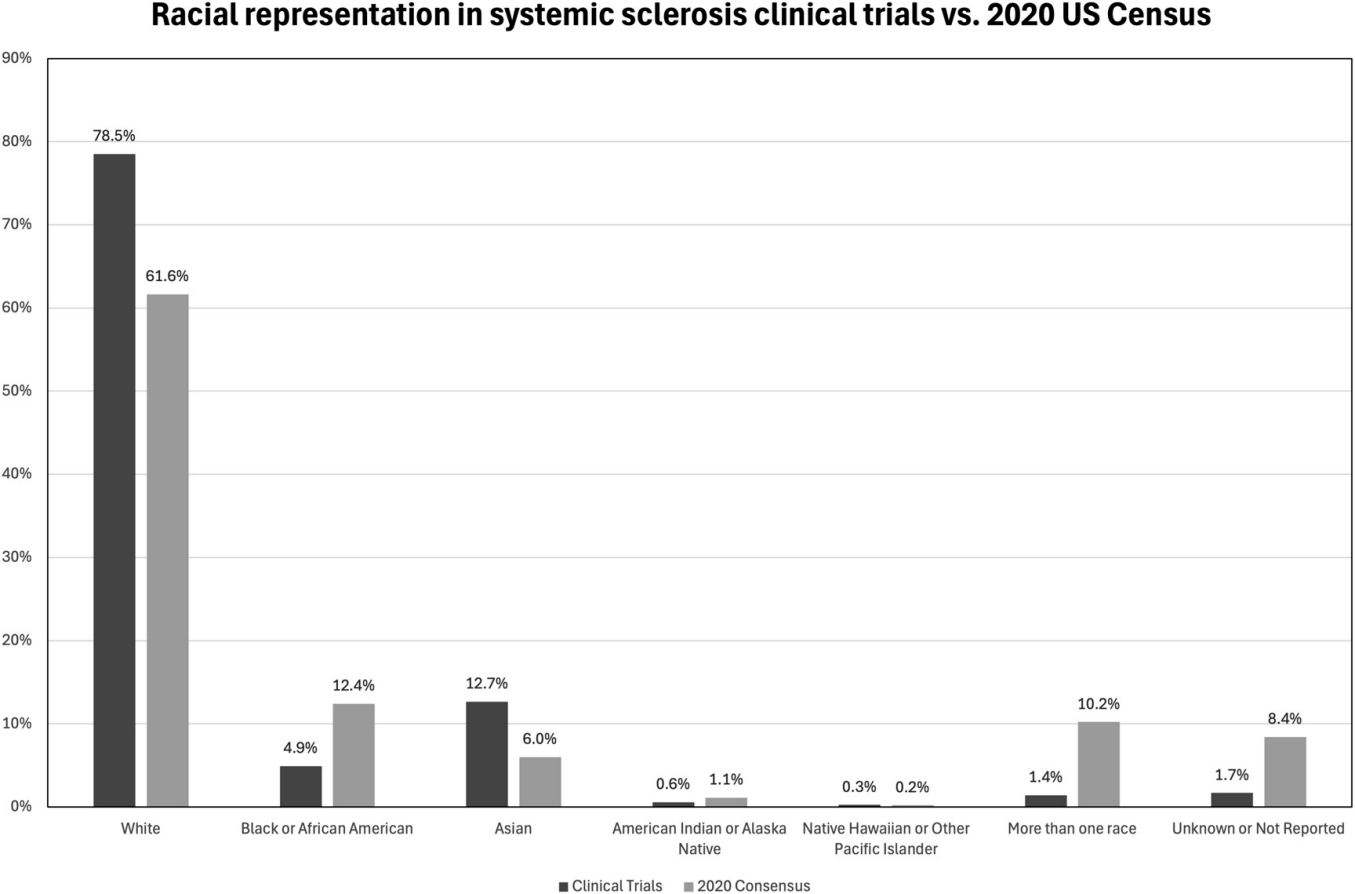
Racial representation in systemic sclerosis clinical trials vs 2020 US Census.

White participants were overrepresented in trials (78.5% vs 61.6%; +16.9 percentage points), as were Asian participants (12.7% vs 6.0%; +6.7 points). Black or African American individuals were markedly underrepresented (4.9% vs 12.4%; −7.5 points). Representation was lower for American Indian or Alaska Native participants (0.6% vs 1.1%; −0.5 points) and substantially lower for those reporting more than 1 race (1.4% vs 10.2%; −8.8 points). Native Hawaiian or Other Pacific Islander representation was similar (0.3% vs 0.2%; +0.1 points). Unknown/not reported constituted 1.7% of trial participants vs 8.4% in Census tabulations (Fig 5).

**Figure 6:**
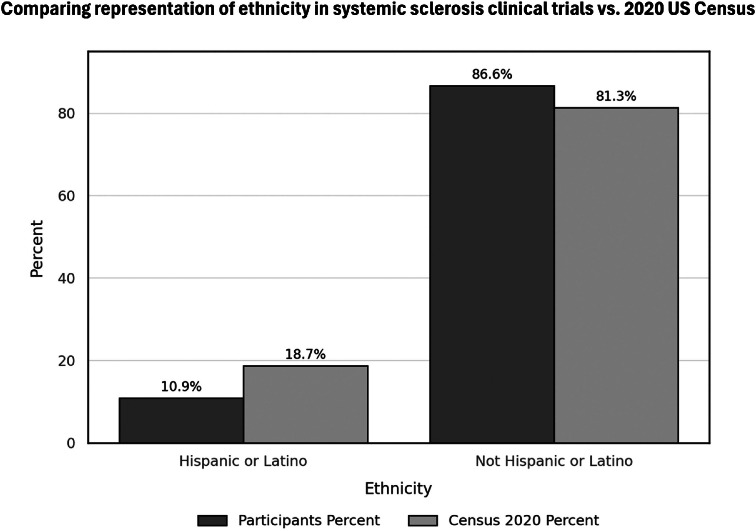
Ethnicity represenation in systemic sclerosis clinical trials vs 2020 US Census.

Regarding ethnicity, Hispanic or Latino individuals represented just 10.9% of participants with known ethnicity status, falling below their 18.7% share in the general population, with a deficit of 7.5 percentage points. Conversely, non-Hispanic participants were overrepresented at 86.6%, compared with 81.3% in the Census (Fig 6).

## DISCUSSION

This analysis confirms that significant racial and ethnic disparities persist in the SSc clinical trial landscape. Racial minority groups remain underrepresented relative to both their disease burden and their population proportions. White participants accounted for the vast majority of enrolled patients; in an observational study of PubMed-indexed clinical trials reporting race, approximately 79% of participants were White, whereas only approximately 6% were Black/African American and 7% Asian [6]. We observe similar findings in our cohort, with White patients being greatly overrepresented than the general population, while Black and Hispanic/Latino individuals were markedly underenrolled.

Several real-world SSc trials illustrate these representation gaps and hint at their underlying causes. For example, the SCOT trial (Scleroderma: Cyclophosphamide or Transplantation), a pivotal study of stem cell transplantation vs cyclophosphamide in treating severe SSc, enrolled 75 patients across US centres [7]. Notwithstanding its importance, the SCOT reports documented approximately 80% White participation while providing limited, nonprominent reporting of race and ethnicity [6]. Similarly, the international *focuSSced* trial spanned 20 countries, yet most of its investigative sites were in North America and Europe. As a result, the overall cohort in *focuSSced* remained mostly White and included very few Black or Hispanic patients [8]. Another example is the Abatacept Systemic Sclerosis Trial (ASSET), a National Institute of Health- and industry-supported multicenter trial in early diffuse cutaneous SSc. Although labelled international, the ASSET trial’s sites were largely confined to the United States and Canada (with a single site in the United Kingdom), which limited its ability to recruit participants outside of those predominantly White patient populations [9]. These cases underscore how trial location and design can influence the diversity of enrolled subjects. Studies centred in Western countries or at tertiary care centres often struggle to reflect the demographic diversity of the broader SSc patient community.

Ensuring demographic and biological representativeness in late SSc trials is not only an equity imperative but also a prerequisite for regulatory-grade evidence that can support approval decisions. SSc is clinically heterogeneous, with differences in sexdistribution (male vs female), organ involvement, and autoantibody profiles that are variably expressed across ethnic groups [2,3,10]. Because targeted therapies may exert differential effects across these subsets, it is critical that late-stage trials enrolled participants representing these phenotypes, sexes/genders, and autoantibody groups. Parallel translational and preclinical programmes, coupled with prespecified subgroup analyses in clinical trials, are needed to define the biological basis of heterogeneity and to detect genuine treatment-by-subgroup interactions that inform precision use.

Multiple factors likely contribute to the ongoing underrepresentation of minority groups in SSc trials. First, the reporting of race and ethnicity data has been largely optional or inconsistent. Nearly half of the trials in our dataset did not report any participant demographic breakdown, an omission that severely limits transparency and the ability to identify disparities. Without uniform requirements, investigators may omit these data, and any diversity gains (or losses) over time remain difficult to quantify. Second, the geographic distribution of trial sites plays a critical role in determining who enrols. The majority of SSc trials have been concentrated in North America and Western Europe, regions that, while having significant SSc research infrastructure, do not capture the full global diversity of patients with SSc. Regions such as Africa, Latin America, and parts of Asia accounted for only a small fraction of trials in our analysis. This imbalance in trial location means that patients from areas with large Black, Hispanic, or other minority populations have had fewer opportunities to participate, thereby skewing enrolment towards White patients from high-income countries. Third, socioeconomic barriers further exacerbate these issues, as trials requiring frequent travel to specialty centres inherently favour patients with the time and resources to participate, introducing a selection bias that correlates with race and income [11]. Together, these factors form a complex backdrop that perpetuates the low enrolment of racial and ethnic minorities in SSc trials.

The implications of this underrepresentation and inconsistent reporting are considerable. A lack of diversity in trials can limit the generalizability of findings and hinder subgroup analyses that might reveal important differences in treatment response or safety. For instance, certain SSc manifestations and outcomes vary by race, but without sufficient inclusion of these groups in clinical studies, it is difficult to determine whether emerging therapies are equally effective across populations. Inconsistent collection of demographic data further compounds this problem, as it prevents researchers from even assessing disparities in many cases. This opacity can mask potential gaps in a therapy’s efficacy or adverse effect profile for minority patients, posing a risk that approved treatments will have unrecognised variability in benefit. Moreover, failing to report and address representation gaps carries ethical and trust repercussions, since patients from underrepresented communities may justifiably question whether a given therapy, developed and tested primarily on White populations, will work for them, potentially reducing confidence and uptake. In short, the current state of SSc trials, with incomplete inclusion of minority groups, threatens to both bias our scientific understanding of SSc and widen existing health outcome disparities if new treatments are not evaluated in the populations most affected by the disease.

Looking ahead, a more concerted effort to improve representation in SSc trials is needed to ensure both equity and scientific rigour. Recent years have seen increasing recognition of this issue at the policy level. For instance, the NIH now requires investigators to outline plans for recruiting women and minority participants in trial grant applications, and the US Food and Drug Administration has issued guidance encouraging broader eligibility criteria and inclusive recruitment strategies in clinical studies [12]. While such measures are promising, their impact will depend on robust implementation in the SSc research arena. Practical strategies to enhance diversity in trials have been proposed, including opening trial sites in community hospitals that serve underrepresented populations, offering remote or local study visits to reduce travel burdens, and engaging with patient advocacy groups to build trust and awareness. The EMPACTA trial serves as a case study in effective inclusion. This phase III trial, conducted during the COVID-19 pandemic, was intentionally designed to enrol mostly minority patients with pneumonia [13]. Its ambitious goal was a significant success, with approximately 85% of the 389 patients were from minority racial and ethnic groups, with a majority Hispanic and significant Black and Native American representation. To achieve this, the trial was conducted in countries and communities heavily affected by COVID-19 but often underrepresented in research, such as Mexico, Brazil, Peru, Kenya, and South Africa, as well as minority-serving hospitals in the United States. Salama et al [13] also engaged local community organisations to build trust, and they provided support to reduce barriers to participation. The outcome was 2-fold: not only did the trial demonstrate the efficacy of tocilizumab in reducing the need for ventilation, but it also proved that a large, diverse trial is achievable on a rapid timeline. The lessons from EMPACTA can inform SSc trials that focused effort, leadership commitment to diversity, and strategic site selection can dramatically shift the demographic makeup of a study. By emulating such models and continuously measuring progress, the SSc research community can gradually close the representation gap.

Ultimately, improving the completeness of demographic reporting and striving for more inclusive enrolment are critical steps for the SSc trial community. By doing so, future trials can provide more reliable subgroup analyses, bolster the generalizability of results, and ensure that advances in SSc therapy development benefit all patient populations equitably.

The validity of these findings depends on the accuracy and completeness of registry records, which frequently omit race/ethnicity data. A central limitation of this study is that much of the epidemiologic evidence in SSc derives from clinical trials in which reporting of race and ethnicity is not mandatory, resulting in incomplete demographic data that constrain inference and external validity. In addition, our reliance on US population benchmarks may not reflect group-specific disease prevalence and is suboptimal for trials with substantial international enrolment. The absence of participant-level data further precludes adjustment for site- and patient-level covariates, while heterogeneity in race classification, such as multiracial and other, limits crosstrial comparability. Incomplete registration, delayed updates, and post hoc revisions also introduce selection and time-lag biases. Finally, as an observational and registry-based analysis, the study is inherently descriptive and cannot establish causal determinants of underrepresentation or evaluate the effectiveness of specific recruitment strategies.

A prominent strength of this study lies in its leveraging a comprehensive, multidecade registry to characterise demographic reporting and representation in SSc trials, using standardised race/ethnicity harmonisation aligned with Office of Management and Budget categories, applying enrolment-weighted pooling to reflect participant volume, and benchmarking representation against US Census data with clear disparity metrics. Finally, our approach is transparent and reproducible from publicly available data, enhancing policy relevance and auditability.

## Conclusion

SSc clinical trials registered between 1999 and 2025 demonstrate significant gaps in racial and ethnic representation and incomplete demographic reporting. These deficiencies threaten the generalizability and equity of emerging therapies. Achieving representative participation in rare disease research is both a scientific imperative and an ethical obligation. Targeted reforms across funding, policy, infrastructure, and community engagement are needed to ensure that all populations benefit equitably from advances in SSc research.
